# Bi-Directional Relationships Between Psychological Symptoms and Environmental Factors in Early Adolescence

**DOI:** 10.3389/fpsyt.2020.574182

**Published:** 2020-09-03

**Authors:** Ziyan Huang, Kaori Endo, Syudo Yamasaki, Shinya Fujikawa, Shuntaro Ando, Mariko Hiraiwa-Hasegawa, Kiyoto Kasai, Atsushi Nishida, Shinsuke Koike

**Affiliations:** ^1^Center for Evolutionary Cognitive Sciences, Graduate School of Art and Sciences, University of Tokyo, Tokyo, Japan; ^2^Department of Medical Science, Graduate School of Medicine, University of Tokyo, Tokyo, Japan; ^3^Department of Psychiatry and Behavioural Sciences, Tokyo Metropolitan Institute of Medical Science, Tokyo, Japan; ^4^Department of Neuropsychiatry, Graduate School of Medicine, University of Tokyo, Tokyo, Japan; ^5^Department of Evolutionary Studies of Biosystems, School of Advanced Sciences, SOKENDAI (The Graduate University for Advanced Studies), Kanagawa, Japan; ^6^University of Tokyo Institute for Diversity and Adaptation of Human Mind (UTIDAHM), Tokyo, Japan; ^7^University of Tokyo Center for Integrative Science of Human Behavior (CiSHuB), Tokyo, Japan; ^8^The International Research Center for Neurointelligence (WPI-IRCN), Institutes for Advanced Study (UTIAS), Tokyo, Japan

**Keywords:** child, young adolescent, cohort, longitudinal analysis, bifactor analysis, structural equation modeling

## Abstract

**Aim:**

Bi-directional relationships between various environmental factors and psychological symptoms can be seen from childhood to adolescence; however, there has been little prospective cohort study, which investigated the relationships simultaneously. In this study, we first distinguished specific psychological symptoms from general psychopathology using bifactor modeling and then tested the relationships between psychological symptoms and environmental factors from childhood to early adolescence using a structural equation model (SEM).

**Methods:**

The analyses were based on Tokyo TEEN Cohort (TTC) data collected between October 2012 and March 2016. We obtained self-reported psychological symptoms and environmental factors from both parents and children (at their ages of 10 and 12). Participants were 3,171 children aged 10 [girls = 1,487 (46.9%), mean age, SD = 10.2, 0.28] and subsequently 12 (N = 3,007, follow-up rate 94.8%, mean age, SD = 12.2, 0.31) from three municipalities in Tokyo area.

**Results:**

The best-fit symptom models included four unique factors and general psychopathology as the common factor. Combining the good fit bifactor model and the SEM, positive relationships between symptoms and environmental factors at the same waves and some bi-directional relationships were found. Especially, general psychopathology at age 10 was associated with bullying at age 12 and parental depressive symptoms at age 10 with general psychopathology at age 12. However, some negative relationships such as bullying/bullied involvement and later psychological symptoms were also seen.

**Conclusion:**

By using the newly introduced methodology, our results were partly consistent with previous literature. Further studies are needed to validate this methodology and accelerate the findings regarding the emergence of psychological symptoms and the impact of environmental factors from childhood to early adolescence.

## Introduction

Environmental influences are known to be the key factors for the emergence of psychological symptoms and psychiatric disorders (1, 2). Clinical and population-based investigations have shown that stressful experiences from childhood to adolescence are common risk factors for the onset of psychiatric disorders in their later lives (3, 4). For example, bully victimization increases the odds of the emergence of depressive episodes (5–7), anxiety (8), and psychotic disorders (9) in later life. These relationships were also observed for psychological symptoms in the general population (10–12).

Parental depressive symptoms (13, 14) and parenting style (15) are also key factors for children’s mental health. A cohort study from U.S. NESARC (n = 43,093) showed that a history of physical, emotional, or sexual abuse in the first 17 years of life was associated with both internalizing and externalizing psychopathology in adults (16). However, there are limited researches exploring the relationships between multiple environmental factors and psychological symptoms simultaneously in a prospective cohort study. Testing such relationships simultaneously could provide additional information about whether a symptom was indeed driven by a specific environmental factor and vice versa. This will also provide a better understanding of the emergence and progression of psychopathology during adolescence (16, 17).

Since the presence of one psychological symptom often comes with others that belong to another clinical category, the potential comorbidity of psychiatric disorders and psychological symptoms should be considered in testing multiple psychological symptoms (18, 19). Bifactor modeling has become a popular method to differentiate specific symptoms from common general psychopathology based on the presence of other psychological symptoms (20–24). A UK ROOTs study (n = 1,159) applied this model for self-reported depression and anxiety at age 14 years and distinguished the specific factors of hopelessness, restlessness, and general worrying by introducing a general factor (23). Another study using a functional magnetic resonance imaging technique showed that there were strong associations between the latent variables of anxiety and amygdala-related connectivity, and between that of irritability and insular-related connectivity (24). In addition, the general psychopathology, namely, the p factor, which is extracted from a bifactor model with various symptoms, was reported and well-replicated (20, 22). However, no prospective cohort study has investigated the relationships between environmental factors and general psychopathology factors and other psychological factors.

In this study, we intended to investigate the relationships between multiple environmental factors and psychological symptoms simultaneously in Tokyo teen cohort (TTC), a population-based prospective cohort in Tokyo, Japan (25, 26). In this cohort, we measured a variety of psychological symptoms such as depressive symptoms and psychotic experiences from children at their age of 10 and 12 and internalized and externalized behavioral problems such as anxiety, somatoform, and aggressive behaviors from their main caregivers. Various environmental factors such as parental depressive symptoms, parenting style, and bullying/bullied involvement at the two waves were also measured. These features of the TTC database enabled us to test the associations between environmental factors and psychological symptoms evaluated by bifactor modeling using structural equation modeling (SEM). We hypothesized that after controlling the correlations between psychological symptoms and environmental factors, general psychopathology at age 10 would have a relationship with some environmental factors at age 12 and vice versa.

## Methods

### Participants

The sample of TTC was recruited from three municipalities (Setagaya-ku, Mitaka-shi, and Chofu-shi) in the metropolitan area of Tokyo (25, 26). The data were obtained between 2012 and 2015 for wave 1 (N = 3,171, girls = 1,487, age: mean [SD] = 122.1 [3.3] months; **Table 1**) and between 2014 and Mach 2017 for wave 2 (N = 3,007, girls = 1,418, age: 146.0 [3.7] months; follow-up rate 94.8%).

**Table 1:** Descriptive statistics of TTC at ages 10 and 12.

		Age 10 (n = 3,171)		Age 12 (n = 3007)		Cohen’s D^a^	p^a^
		Number/*mean*	%/*SD*	Number/*mean*	%/*SD*		
Age (month)		*122.1*	*3.3*	*146.0*	*3.7*		
Sex (female)		1,487	46.9%	1,418	47.2%		
Psychological symptoms							
CBCL	Internalized score	*53.46*	*8.72*	*52.16*	*8.8*	0.18	<0.01
	Externalized score	*51.41*	*8.50*	*49.65*	*8.24*	0.28	<0.01
SDQ	Emotional symptom	*1.21*	*0.55*	*1.17*	*0.50*	0.06	<0.01
	Conduct problems	*1.21*	*0.54*	*1.19*	*0.52*	0.04	0.03
	Hyperactivity/inattention	*1.23*	*0.59*	*1.17*	*0.51*	0.11	<0.01
	Peer relationship	*1.17*	*0.51*	*1.18*	*0.52*	0.02	0.28
	Prosocial behavior	*1.43*	*0.71*	*1.5*	*0.76*	0.10	<0.01
SMFQ		*4.76*	*4.58*	*3.84*	*4.49*	0.17	<0.01
APSS		*0.87*	*0.91*	*0.83*	*0.99*	0.03	0.12
Bully involvement							
Bullied	Left out	492	16.1%	213	8.6%	0.17	<0.01
	Called mean names	595	19.5%	260	10.5%	0.19	<0.01
	Hit lightly	351	11.5%	130	5.2%	0.15	<0.01
	Hit strongly	194	6.3%	42	1.7%	0.18	<0.01
	Things taken	76	2.5%	35	1.4%	0.06	<0.01
	Not bullied	2081	68.0%	2022	81.3%	0.23	<0.01
Bullying	Left out	21	7.0%	113	4.5%	0.06	<0.01
	Called mean names	184	6.1%	98	3.9%	0.08	<0.01
	Hit lightly	133	4.4%	60	2.4%	0.07	<0.01
	Hit strongly	42	1.4%	10	0.4%	0.08	<0.01
	Things taken	15	0.5%	7	0.3%	0.02	0.44
	Not bully	2584	86.1%	2273	90.9%	0.10	<0.01
Parental depressive symptoms							
K6		*2.94*	*3.33*				
GHQ-28	Depression-related 7 items				*8.04*	*4.28*	
Educational level of father							
High school or less		542	18.0%				
2-year college		409	13.6%				
4-year university		1692	56.1%				
Graduate university		374	12.4%				
Educational level of mother							
High school or less		524	16.7%				
2-year college		1391	44.2%				
4-year university		1126	35.8%				
Graduate university		105	3.3%				
Annual household income (10 000 yen)							
0–299		142	4.7%				
300–599		763	25.0%				
600–999		1224	40.2%				
1000+		917	30.1%				

CBCL, the child behavior checklist; SDQ, the strength and difficulties questionnaire; SMFQ, the short mood and feeling questionnaire; APSS, the adolescent psychotic-like symptom screener; K6, the Kessler psychological distress scale; GHQ-28, the 28-item version of the general health questionnaire.

^a^Paired t-test was performed between age 10 and 12.

There were no statistical differences in demographic characteristics including age, sex, or paternal and maternal education between those who took part in the followed-up study and those who did not (*p* > 0.05). Ethical approval for this study was obtained from the Ethical Committee of Tokyo Metropolitan Institute of Medical Science (number: 12-35), the University of Tokyo (number: 10057), and SOKENDAI (the Graduate University for Advanced Studies, number: 2012002). The children’s main caregivers (usually mothers) gave written informed consent. The data analysis was conducted between September 2018 and November 2019.

### Surveys

We used TTC child and parent self-report questionnaires for environmental factors and psychological symptoms at both wave 1 and 2. 98.3 and 97.9% of the respondents (main caregivers) were the participants’ mothers in wave 1 and 2, respectively. All the examiners had ensured anonymity to all participants before they gave any responses. In order to maintain confidentiality and reliability of the sensitive questions, the printed questionnaires [the child behavior checklist (CBCL), the general health questionnaire (GHQ-28), socio-economic status, the short mood and feeling questionnaire (SMFQ), the adolescent psychotic-like symptom screener (APSS), and bullying involvement] were all put in concealed envelopes. In addition, the participants were also informed that a third person would be assigned to code their responses.

### Psychological Symptom Measurement

For psychological symptoms, CBCL (27) and the strength and difficulties questionnaire (SDQ) (28) were obtained from their main caregivers, while SMFQ (29) and APSS (30) were from the children at both waves.

### Environmental Factor Assessment

Environmental factors such as bullying/bullied involvement and warm parenting style were obtained from the caregivers. In addition, bullying/bullied involvement was also assessed by the children themselves. Parental depressive symptoms were assessed using the Kessler psychological distress scale (K6) (31) at wave 1 and the 28-item version of GHQ-28 (32) at wave 2. Socio-economic status at the children’s age 10 were indicated by their annual household income and parental educational attainments from the parental questionnaire (25). Life satisfaction consisted of four items reported by the children at both waves.

### Statistical Analysis

The psychological assessment of each survey was tested by an exploratory bifactor analysis using ‘psych’ package (33) of R version 3.4.4 (34). A bifactor model has a bi-factor structure, composed of a general factor and several group factors, which can provide a common and specific psychological symptom structure for the dataset (35). We applied a bi-quartimin rotation (36) to fix the correlations between the general factor with the group factors at zero but at the same time allowed non-zero correlations between the group factors. Numbers of factors were determined using a scree test (37). The final number of factors to be retained depended on whether the factors have at least three items with 0.40 or higher factor loadings. After we determined the number of factors, we further deleted items with small factor loadings to get a good fit while allowing some factor loadings to have smaller than 0.40 in the same factor structure.

Confirmatory factor analysis was performed using ‘lavaan’ package (38). The model was estimated by the maximum likelihood method and the missing values were handled by full information maximum likelihood. The criteria of a fitted model were defined by p ≥ 0.05 in a chi-square test, or root mean square error of approximation (RMSEA) ≤ 0.05 and comparative fit index (CFI) ≥ 0.90. For the environmental factors, we used exploratory factor analysis followed by confirmatory factor analysis, with the same steps.

Finally, SEM using ‘lavaan’ package was conducted to explore the associations and relationships between environmental factors and psychological symptoms from wave 1 to wave 2 in one model. The paths of relationships were set under time course. Non-significant paths (*p* > 0.1) were removed, and covariance was set to zero. We calculated the models until the coefficient of every path and covariance’s *p* value become less than 0.05.

## Results

### Demographic Characteristics

All psychological symptom scale scores decreased in 2 years except for the peer relationship sub score in the SDQ and the APSS score (*p’s* < 0.05, **Table 1**). Similarly, bullying/bullied involvement, except for “taking others’ things”, decreased significantly. Boys at age 10 had greater psychological symptoms in the SDQ, SMFQ, and APSS scores compared to girls, but such difference became non-significant in some scores at age 12 (**Tables S1** and **S2**). Similar trends of gender difference were seen in bullying/bullied involvement, but girls had greater bullying/bullied involvement for ‘left out’ compared to boys at age 12. Most of the correlations between psychological symptoms were significant in both waves (**Figure S1**).

### Bifactor Models for Psychological Symptoms

**Figure 1:**
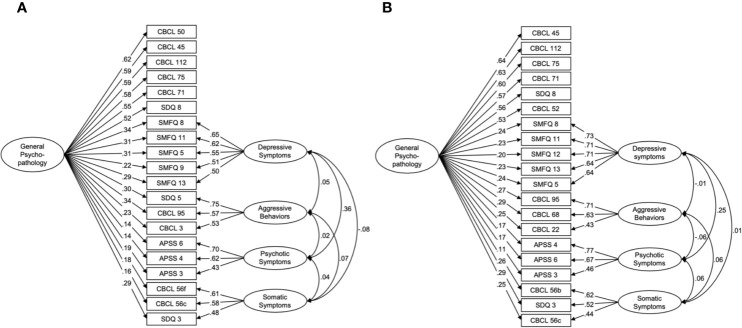
Bifactor models for psychological symptoms at ages 10 and 12. The best fit models at ages 10 **(A)** and 12 **(B)** include unique factors of depressive symptoms, aggressive behaviors, psychotic symptoms, and somatic symptoms, as well as a common factor of general psychopathology. CBCL, the child behavior checklist; SDQ, the strength and difficulties questionnaire; SMFQ, the short mood and feeling questionnaire; APSS, the adolescent psychotic-like symptom screener.

For psychological symptoms, data from the two waves both suggested a five-factor structure that comprised a general psychopathology factor and four unique factors (depressive symptoms, aggressive behaviors, psychotic symptoms, and somatic symptoms; age 10: *χ^2^* = 787.5, *df* = 150, *p* < 0.001, *CFI* = 0.953, *RMSEA* = 0.037, **Figure 1A**, **S2A** and **Table S3**; age 12: *χ^2^* = 639.6, *df* = 150, p < 0.001, *CFI* = 0.962, *RMSEA* = 0.033; **Figure 1B**, **S2B** and **Table S4**).

The models consisted of 20 items at both ages; 15 items were common in both models (**Tables S3** and **S4**, respectively). Six items were specific to general psychopathology related to the internalized problems of anxiety from the CBCL and SDQ. Depressive symptoms, aggressive behaviors, psychotic symptoms, and somatic symptoms consisted of five items from the SMFQ, three items from the CBCL and SDQ, three items from the APSS, and three items from the CBCL and SDQ, respectively.

### Factor Analyses for Environmental Factors

Wave 1 data appeared to support a six-factor structure which consisted of the child’s life satisfaction, bullied, bullying, parental depressive symptoms, warm parenting style, and socioeconomic status (*χ^2^* = 800.8, *df* = 194, *p* < 0.001, *CFI* = 0.963, *RMSEA* = 0.031; **Figure S3A** and **Table S5**). Wave 2 data supported a five-factor structure that contained the same latent variables as wave 1 except the socio-economic status factor (*χ^2^* = 844.6, *df* = 192, *p* < 0.001, *CFI* = 0.960, *RMSEA* = 0.041; **Figure S3B** and **Table S6**).

### Relationships Between Psychological Symptoms and Environmental Factors in One Model

**Figure 2:**
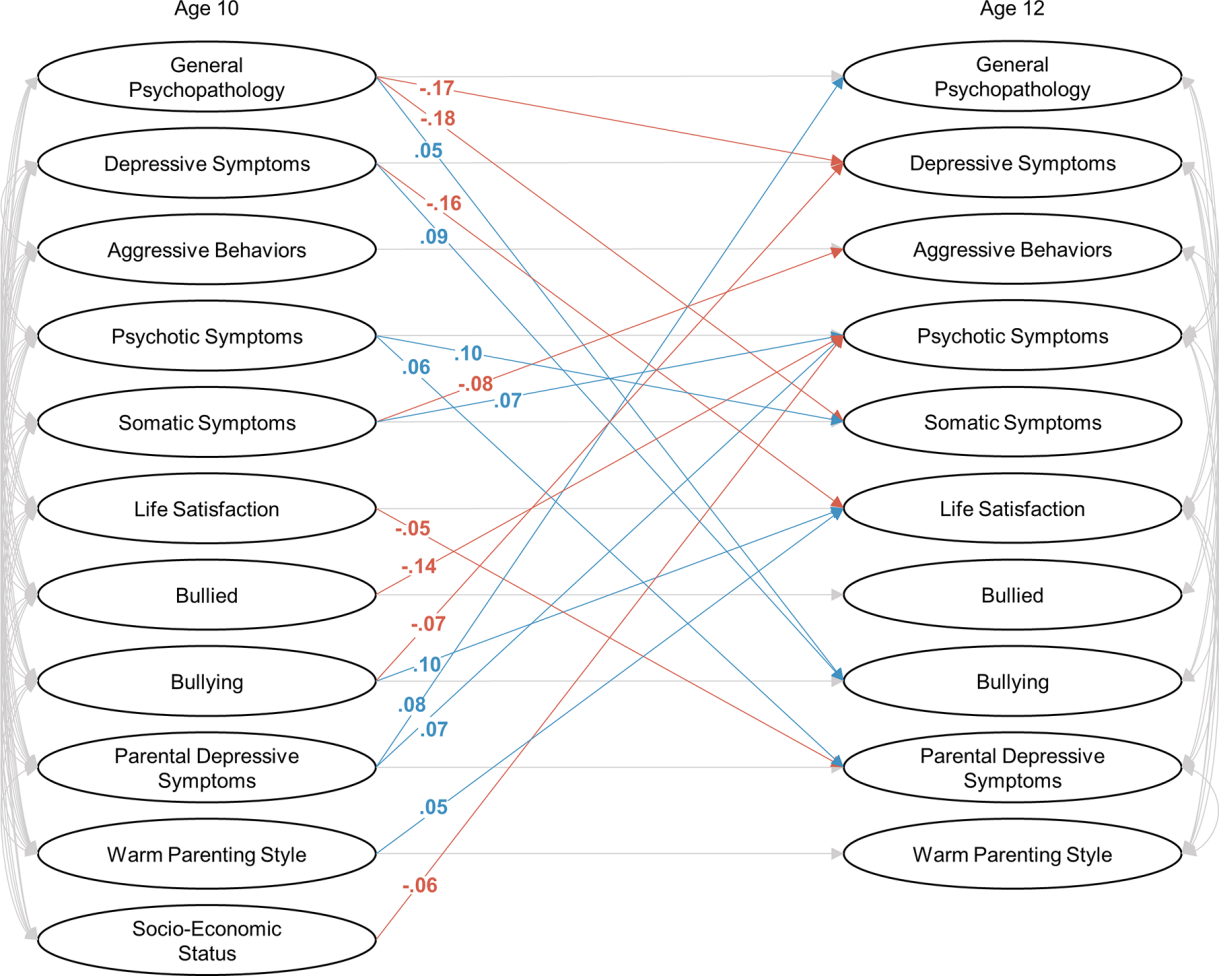
Bifactor models combining with a structural equation modeling at ages 10 and 12. Significant positive and negative relationships between one and another factor were shown in blue and red, respectively. The correlations and relationships in one factor between ages 10 and 12 were shown in gray, and standardized coefficients were shown in **Figures 3** and **4**.

**Figure 3:**
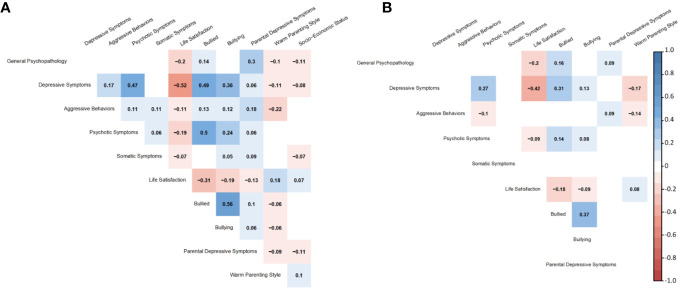
Standardized coefficients of correlations between latent variables at ages 10 **(A)** and 12 **(B)**.

**Figure 4:**
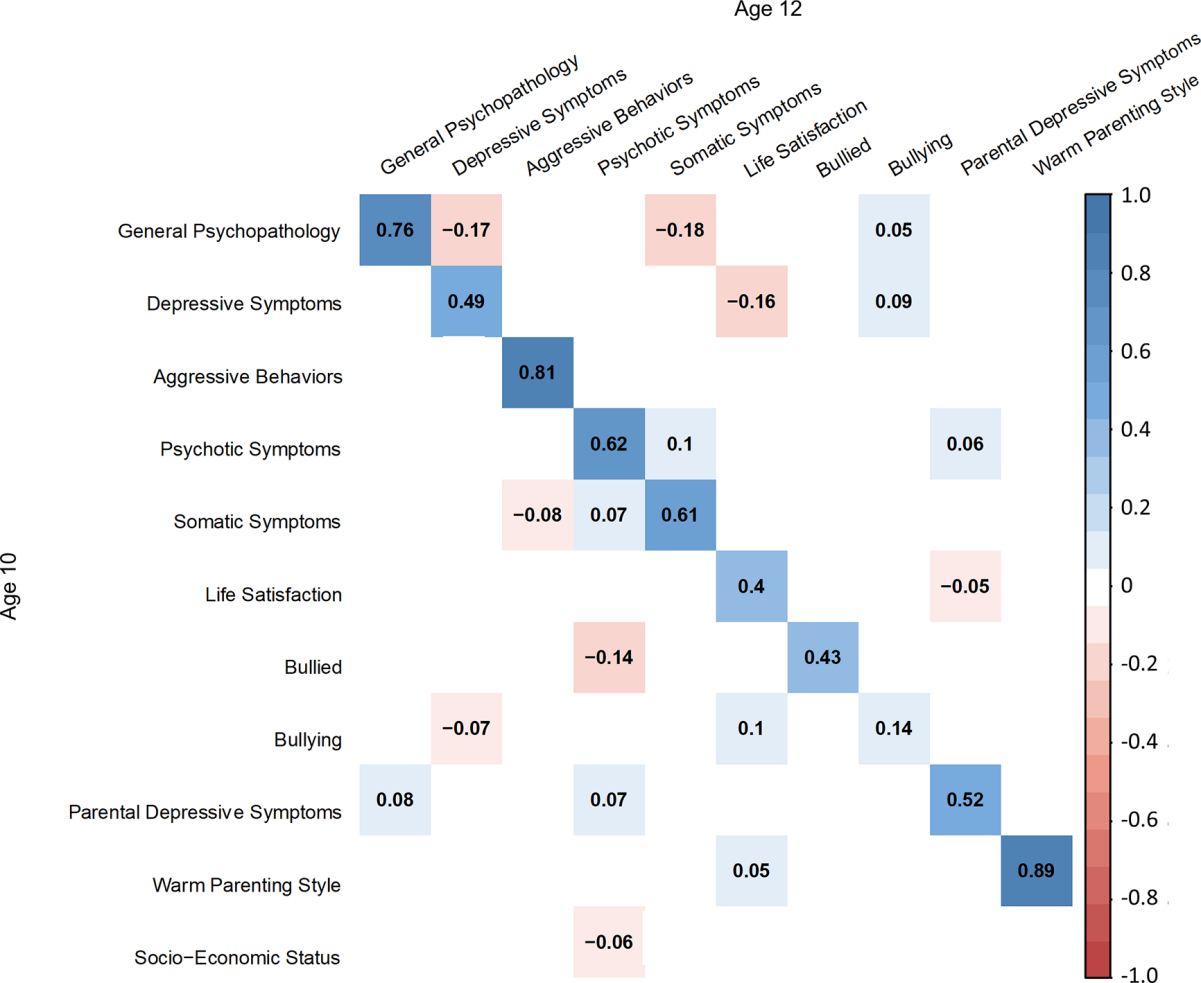
Standardized coefficients of regression between latent variables at ages 10 and 12.

The latent structures of the psychological symptoms (bi-factor models) and the environmental factors had a good fit in a SEM model (*χ^2^* = 10371.94, *df* = 3292, *p* < 0.001, *CFI* = 0.907, *RMSEA* = 0.026; **Figures 2**–**4**). Each symptom and environmental factor at age 12 was mostly influenced by the correspondent symptom and factor at age 10 (*β* = 0.14 ~ 0.89, *p’s* < 0.01; **Figure 4**).

Some correlations between factors were significant at age 10 but became non-significant at age 12 (**Figures 2** and **3**): symptom-symptom correlations between depressive symptoms and aggressive behaviors (*β* = 0.17, *SE* = 0.02, *p* < 0.01 at age 10, the same below), aggressive behaviors and somatic symptoms (*β* = 0.11, *SE* = 0.03, *p* < 0.01), and psychotic symptoms and somatic symptoms (*β* = 0.06, *SE* = 0.03, *p* = 0.03); symptom-environmental correlations between general psychopathology and warm parenting style (*β* = -0.10, *SE* = 0.02, *p* < 0.01), depressive symptoms and parental depressive symptoms (*β* = 0.06, *SE* = 0.02, *p* < 0.01), aggressive behaviors and life satisfaction (*β* = -0.11, *SE* = 0.02, *p* < 0.01), bullied (*β* = 0.13, *SE* = 0.02, *p* < 0.01), bullying (*β* = 0.13, *SE* = 0.03, *p* < 0.01), psychotic symptoms and parental depressive symptoms (*β* = 0.06, *SE* = 0.02, *p* = 0.02), somatic symptoms and life satisfaction (*β* = -0.07, *SE* = 0.02, *p* < 0.01), bullying (*β* = 0.05, *SE* = 0.03, *p* = 0.04), and parental depressive symptoms (*β* = 0.09, *SE* = 0.02, *p* < 0.01); environmental-environmental correlations between life satisfaction and parent depressive symptoms (*β* = 0.05, *SE* = 0.03, *p* = 0.04), bullied and parent depressive symptoms (*β* = 0.10, *SE* = 0.02, *p* < 0.01) and warm parenting style (*β* = -0.06, *SE* = 0.02, *p* < 0.01), bullying and parent depressive symptoms (*β* = 0.06, *SE* = 0.02, *p* = 0.01), warm parenting style (*β* = -0.06, *SE* = 0.02, *p* = 0.02), and parent depressive symptoms and warm parenting style (*β* = -0.09, *SE* = 0.02, *p* < 0.01).

The inter-factor relationships between the psychological symptoms showed that general psychopathology at age 10 was negatively associated with depressive and somatic symptoms at age 12 (*β* = -0.17, *SE* = 0.05, *p* < 0.01; *β* = -0.18, *SE* = 0.06, *p* < 0.01; respectively). Psychotic and somatic symptoms were associated with each other (psychotic to somatic: *β* = 0.10, *SE* = 0.03, *p* < 0.01; somatic to psychotic: *β* = 0.07, *SE* = 0.03, *p* = 0.01). Somatic symptom at age 10 was negatively associated with aggressive behaviors at age 12 (*β* = -0.08, *SE* = 0.03, *p* < 0.01).

The relationship between environmental factors and psychological symptoms revealed that parental depressive symptom at children’s age 10 was positively associated with general psychopathology (*β* = 0.08, *SE* = 0.02, *p* < 0.01) and psychotic symptom at age 12 (*β* = 0.07, *SE* = 0.02, *p* < 0.01). Bullying at age 10 was negatively associated with bullied with psychotic symptom (*β* = -0.14, *SE* = 0.03, *p* < 0.01) and depressive symptom at age 12 (*β* = -0.07, *SE* = 0.03, *p* < 0.01). Lower socio-economic status was associated with psychotic symptoms at age 12 (*β* = -0.06, *SE* = 0.03, *p* < 0.01).

For symptom to environment paths, life satisfaction at age 12 was affected by depressive symptom (*β* = -0.16, *SE* = 0.03, *p* < 0.01), parenting style (*β* = 0.05, *SE* = 0.02, *p* = 0.01), and bullying (*β* = 0.10, *SE* = 0.03, *p* < 0.01) at age 10. Bullying at age 12 was also affected by general psychopathology (*β* = 0.05, *SE* = 0.03, *p* = 0.04) and depressive symptom (*β* = 0.09, *SE* = 0.03, *p* < 0.01) at age 10. Parental depressive symptoms at age 12 was affected by psychotic symptom (*β* = 0.06, *SE* = 0.02, *p* = 0.01) and life satisfaction (*β* = -0.05, *SE* = 0.02, *p* = 0.01) at age 10.

## Discussion

In this study, we investigated the relationships between multiple psychological symptoms and environmental factors simultaneously using a bifactor model in combination with an SEM approach in a population-based cohort from childhood to early adolescence. Apart from the positive relationships between psychological symptoms and environmental factors at the same waves, there were bi-directional relationships between them. Especially, we found a relationship between general psychopathology at age 10 and bullying behavior at age 12, and between parental depressive symptoms at age 10 and general psychopathology at age 12. Unexpected results were also seen, for instance, bullying/bullied involvement was negatively associated with later symptoms. To the best of our knowledge, this is the first study that showed the bi-directional relationships between multiple psychological symptoms and environmental factors in one statistical model.

In line with previous studies, we found significant correlations between psychological symptoms and environmental factors at both waves. Comparing the two waves, correlation coefficients between symptoms and environmental factors decreased overall and some correlations became non-significant from age 10 to 12. The reduction in correlations could be due to an increase of specific psychopathologies which may become more differentiable from other symptoms. In addition, the child-parent relationship could change during this period (39) and parental response pattern for child behaviors and symptoms may also change. Parental responses could be influenced by parental mental state and subjective evaluation of their children, leading to an over/under-evaluation. A decrease in correlations was also seen among environmental factors. For example, bully involvement was correlated with parental depressive symptoms and parenting style at children’s age 10, while the correlations became non-significant at 12. This may be caused by a shift of children’s significant other from parents to peer, and hence the child-parent relationship becomes less influential (40).

After examining the correlations between psychological symptoms and environmental factors at each wave, we found that general psychopathology at age 10 related to bullying behavior at age 12 and parental depressive symptoms at age 10 related to general psychopathology at age 12. According to the comparative studies between Japan and England, Japanese involvement in bullying tended to happen more in “friends’ group” and by more numbers of bullying persons (41). Bifactor analyses showed that general psychopathology factor in this study mainly consisted of anxiety-related symptoms from parental questionnaires at both waves, and therefore, it is possible that highly anxious students involve more in bullying because they are anxious about being bullied themselves. Also, the influence of parental depressive symptom to child’s mental states is consistent with previous literature (13, 14). Our results strengthen the view that close attention should be paid to parental mental state in order to prevent the development of general psychopathology in children. Still, another possible explanation for this finding is that parental mental state may influence their recognition of the value in their children. Future studies should use more reliable evaluation of anxiety and depression in the combination of psychological interviews and multi-dimensional reports (i.e. child, parents, and teachers).

Unexpectedly, we found that bullying/bullied involvement was negatively associated with later psychological symptoms, which was contrary to our initial hypothesis. A number of studies revealed that bullying/bullied involvement has a strong impact on future emergence of psychological symptoms and psychiatric disorders (5–7, 17, 42, 43). One possible explanation for this finding may be that the awareness and experience of bullying/bullied in their primary schools may rather support the decrease of psychological symptoms in their junior high schools. During the waves, the participants moved into junior high schools, and therefore, their school environment and peer relationship changed dramatically. This awareness and experiences may have strengthened their psychological resilience toward later symptom emergence.

A decrease in the number of children reported bullied, bullying and psychological symptoms from age 10 to age 12 has frequently been reported, and the former trend was also observed in our study (44–46). A study shared the same target age group with us and is an exceptional case where such a pattern was not observed (47). They have found a positive bidirectional relationship between bullied experience, and depression and anxiety at age 10–11 and 12–13 using path analysis. This may suggest that the change in our sample’s response pattern to the instruments contributes to the unexpected correlations found. Another possibility is that our data was obtained from parental responses for anxiety-related items and both parental and children’s responses in depression and bully-related items. The multi-informant assessment generally increases the validity and reliability of the children’s responses (48), but it would reduce the tendency of positive response. Since early adolescence is a critical period to the validity and reliability of the responses, results obtained regarding the psychological symptoms and bully involvement could considerably change according to who responded the information, future studies considering this will be needed. Compared to this study, pervious research on the impact of bullying/bullied involvement to later psychological symptoms was based on a longer observation period through adolescence (5, 43) and adulthood (6, 7, 17, 42). To investigate the long-term impact of bullying/bullied involvement, we plan to follow-up and obtain data at age 14 and later in TTC. We also plan to perform a stratified analysis to see how each factor changes across adolescent development and further test the relationship between psychological symptoms and environmental factors. It would be interesting to investigate the critical time period or duration of bully involvement, which could lead to development in psychiatric disorders in further longitudinal studies.

There are some limitations in this study. First, as discussed above, we distinguished specific symptoms from general psychopathology; however, there could be response bias included in the general psychopathology factor (49, 50). In this study, parental responses applied to the models more than children’s responses, and the general psychopathology factor could be biased by parental mental condition and response pattern. Second, adolescent physical and psychological development may alter the understandings of items in questionnaires and thus change their responses. Third, a considerable change in physical and psychological development according to the beginning of secondary sex characteristics could alter the pattern of psychological symptoms and environmental factors (51). Gender differences were not specifically investigated in the model since the sample size for each gender is still too small to converge and fit the model. In previous studies, there were relatively small gender differences in symptoms and environmental factors compared to those for adolescence and adulthood. Fourth, as bifactor model approach required a considerable amount of variance in responses, some rare but crucial psychological and environmental items were dropped from model construction such as criminality (52), severe bullying/bullied involvement (7), and abuse (16, 53). Multi-dimensional responses for assessing the severity of the psychological symptoms and risk factors and relatively lower prevalence of them may provide more reliable identification of the cases (54, 55). Thus, further investigations are needed to build a more robust model by combining the statistical approach with more reliable responses.

The present study showed the bi-directional relationships between multiple psychological symptoms and environmental factors from childhood to early adolescence in one model. The correlations and relationships were mostly consistent with previous studies; however, some contradictory relationships to previous findings were also seen. The reason could be partially explained by the change of response pattern for both children and their parents according to adolescent development and their socio-environmental changes in this period. Bifactor modeling combining with an SEM approach enabled us to figure out the unique relationships between psychological symptoms and environmental factors. The results provide a better understanding of the emergence of psychological symptoms and the relationships with environmental factors from childhood to early adolescence.

## Data Availability Statement

The data analyzed in this study is subject to the following licenses/restrictions: Data from TTC is archived in the Tokyo Metropolitan Institute of Medical Science. Collaboration in data analysis and publication will be welcome through specific research proposals sent to the research committee. The initial contact point for collaborations is [nishida-at@igakuken.or.jp]. Requests to access these datasets should be directed to AN, nishida-at@igakuken.or.jp.

## Ethics Statement

The studies involving human participants were reviewed and approved by the Ethical Committee of Tokyo Metropolitan Institute of Medical Science (number: 12-35), the University of Tokyo (number: 10057), and SOKENDAI (the Graduate University for Advanced Studies, number: 2012002). Written informed consent to participate in this study was provided by the participants’ legal guardian/next of kin.

## Author Contributions

ZH and SK designed the work, conducted statistical analyses, and wrote the draft manuscript. KE, SY, SF, SA, AN, and SK contributed to data acquisition and managed the quality of the dataset. All authors contributed to the article and approved the submitted version.

## Funding

This research was supported by the Agency for Medical Research and Development (AMED) under grant numbers JP19dm0307001 and JP19dm0307004 and by Japan Society for the Promotion of Science (JSPS) KAKENHI grant numbers 16H06395, 16H06396, 16H06398, 16H06399, and 19H04878. This study was also supported by the University of Tokyo Center for Integrative Science of Human Behavior (CiSHuB) and the International Research Center for Neurointelligence (WPI-IRCN) at the University of Tokyo Institutes for Advanced Study (UTIAS).

## Conflict of Interest

The authors declare that the research was conducted in the absence of any commercial or financial relationships that could be construed as a potential conflict of interest.
