# Supplementary material for: Bi-Directional Relationships Between Psychological Symptoms and Environmental Factors in Early Adolescence
Source: Front Psychiatry. 2020 Sep 3;11:574182. doi: 10.3389/fpsyt.2020.574182 (PMC7495193; doi:10.3389/fpsyt.2020.574182)
Supplement: Supplementary file 1 [file DataSheet_1.docx]

**Bi-directional relationships between psychological symptoms and environmental factors from childhood to early adolescence**

Ziyan Huang, Kaori Endo, Syudo Yamasaki, Shinya Fujikawa, Shuntaro Ando, Mariko Hiraiwa-Hasegawa, Kiyoto Kasai, Atsushi Nishida, Shinsuke Koike.

**Online-only Supplements**

[Selection of TTC participants from baseline survey 2](#_Toc49684255)

[Psychological symptom measurement 2](#_Toc49684256)

[Child Behavior Checklist (CBCL) 2](#_Toc49684257)

[Strength and Difficulties Questionnaire (SDQ) 2](#_Toc49684258)

[Short Mood and Feelings Questionnaire (SMFQ) 2](#_Toc49684259)

[Adolescent Psychotic-Like Symptom Screener (APSS) 3](#_Toc49684260)

[Environmental factor assessment 3](#_Toc49684261)

[Parental depressive symptom 3](#_Toc49684262)

[Warm parenting style 3](#_Toc49684263)

[Bully involvement 3](#_Toc49684264)

[Life satisfaction 4](#_Toc49684265)

[Socio-economic status 4](#_Toc49684266)

[References 5](#_Toc49684267)

[Table S1. Descriptive statistics of TTC at age 10 between boys and girls 6](#_Toc49684268)

[Table S2. Descriptive statistics of TTC at age 12 between boys and girls 8](#_Toc49684269)

[Table S3. Bifactor analysis of psychological symptoms at age 10 10](#_Toc49684270)

[Table S4. Bifactor analysis of psychological symptoms at age 12 12](#_Toc49684271)

[Table S5. Factor analysis of environmental factors at age 10 14](#_Toc49684272)

[Table S6. Factor analysis of environmental factors at age 12 16](#_Toc49684273)

[Figure S1. Correlation matrix between psychological symptom scores at ages 10 and 12. 18](#_Toc49684274)

[Figure S2. Scree plot of psychological symptoms at ages 10 and 12. 19](#_Toc49684275)

[Figure S3. Scree plot of environmental factors at ages 10 and 12. 20](#_Toc49684276)

# **Selection of TTC participants from baseline survey**

The candidate participants of the Tokyo TEEN Cohort study (TTC) were chosen from 4,478 children who had participated in the Tokyo Early Adolescence Survey (T-EAS) (Ando et al., 2019; Okada et al., 2019). A total of 3,000 children had been set as the target number of participants for the second wave of TTC. During the selection process of the participants, no inclusion criteria were applied although an oversampling method was used in order to compensate for the low follow-up rate of families with low annual household income. Among the children who had participated in T-EAS and showed interest in participating in the cohort study, 620 children, whose household annual income was lower than 4,990 thousand yen, were invited. From the rest of the 3,858 children, 2,551 children were randomly invited to the second wave of TTC.

# **Psychological symptom measurement**

## **Child Behavior Checklist (CBCL)**

The Child Behavior Checklist (CBCL) (Achenbach TM, 1991) for ages 6-18 was a widely used questionnaire from which child’s behavioral problems can be obtained by parents’ responses. It consists of 113 items, 8 subscales, aggressive behavior, anxious/depressed, attention problems, rule-breaking behavior, somatic complaints, thought problems, withdrawn/depressed, and 2 upper scales, internalized problems(anxious/depressed, somatic complaints and withdrawn/depressed) and externalized problems(aggressive behavior and rule-breaking behavior). The responses were given on a 3-point scale: not true =0, somewhat or sometimes true =1, very true or often true =2. In TTC, we collected 79 and 82 items at ages 10 and 12 respectively, and used 18, 10, 9, 7, 7 items related to aggressive behaviors, anxious/depressed, somatic complaints, thought problems, and withdrawn/depressed respectively for the analysis.

## **Strength and Difficulties Questionnaire (SDQ)**

Completed by main care givers, we also get the Strength and Difficulties Questionnaire (SDQ) (Matsuishi et al., 2008), a behavioral screening questionnaire, which consists of 25 items and 5 subscales, each containing 5 items, emotional symptoms, conduct problems, hyperactivity-inattention, peer relationship, and prosocial behaviors. Responses were given on a 3-point scale: not true =0, somewhat or sometimes true =1, very true or often true =2. From SDQ, we included items related to conduct problems and emotional symptoms respectively.

## **Short Mood and Feelings Questionnaire (SMFQ)**

From children, the 13-item Short Mood and Feelings Questionnaire (SMFQ) (Angold, A., Costello, E. J., Messer, S. C., Pickles, 1995) was used to measure their subjective depressive symptoms. Responses were given on a 3-point scale: not true =0, somewhat or sometimes true =1, very true or often true =2.

## **Adolescent Psychotic-Like Symptom Screener (APSS)**

From children, the Adolescent Psychotic-Like Symptom Screener (APSS) (Kelleher, Harley, Murtagh, & Cannon, 2011), which consists of 7 items selected from the Diagnostic Interview Schedule for Children (DISC) (Costello A, Edelbrock C, Kalas R, Kessler M, 1982) and corresponds to psychotic experiences including hallucination and delusion, was used to assess their psychotic experiences. Since there were 5 items (mind reading, TV/radio, spying, auditory hallucinations, visual hallucinations) being recorded at age 12, only scores from these 5 items were subjected to further analyses. Scores were set to no =0, yes, likely =0.5, yes, definitely =1 following the original studies.^6^

# **Environmental factor assessment**

## **Parental depressive symptom**

From the parental self-reported questionnaires, we obtained subjective depressive symptoms from their main caregivers using the K6 scale (Kessler et al., 2002) at age 10 and the 28-item version of the General Health Questionnaire (GHQ-28) (Goldberg & Hillier, 1979) at age 12. In the GHQ-28, we used 7 items that consist of depressive symptom subscale. K6 was scored on a 5-point scale: not at all = 1 to always = 5. GHQ-28 was scored on a 4-point scale: not at all = 1, no or little = 2, yes or once = 3, often = 4.

## **Warm parenting style**

In the parental self-reported questionnaires, we used 5 items for warm parenting style, regarding to talk — “do you often talk with child?”, relationship — “Do you think you are in a good relationship with your child?”, protection — “do you try to protect your child from every difficulty occurred in life?”, telling love — “do you tell your child that you love or cherish him/her?”, and praise — “do you praise your child?” All items were responded using a 4-Likert scale (for talk: no for more than a day=1, once a day =2, about an hour a day = 3, more than 2 hours a day =4; for relationship: very bad = 1, not very good = 2, not bad = 3, very good = 4; for protection, telling love and praise: seldom = 1, sometimes = 2, often = 3, always = 4).

## **Bully involvement**

Bully involvement was assessed using self-reported questions according to the definition of bullying defined in the Olweus Bully/Victims Questionnaire (OBQ) (Solberg & Olweus, 2003). From both child and parent self-reports, we got for the bullying/bullied involvement. From both reports, we got “have you/your child bullied or been bullied in last 2 months inside/outside school?” at age 10, and “have you/your child bullied or been bullied in last 2 months?” at age 12,The participants responded as: several times a week = 5, about once a week = 4, 2 or 3 times a month = 3, once or twice in last 2 month=2, no = 1. We also used detailed questions in the children’s questionnaires: (i) being left out, “Other students left me out of things on purpose, excluded me from their group of friends, completely ignored me, or called mean names in behind;” (ii) called mean names, “I was called mean names, was made fun of, or teased in a hurtful way;” (iii) being hit lightly, “I was hit lightly, pushed or kicked pretending they are just playing;” (iv) being hit strongly, “I was hit, pushed, or kicked strongly;” (v) things being taken, “I had money or other things taken away from me or damaged;” and not bullied/bully, “I didn’t bully.” If there was no check for any six boxes, we regarded their response as NA, otherwise we regarded checked boxes as: yes = 1 and no = 0. We used only first 3 items out of 5 bullying/bullied experiences, because the latter 2 items had low positive responses (Table 1).

## **Life satisfaction**

Four items were used to measure the satisfaction level of the children in themselves for their family members, “Are you satisfied with your family?” yourself, “Are you satisfied with your yourself?” school, “How do you feel about study in school?” and your friend, “How do you feel about your friends?” All responses were scored in a 7-point Likert scale: not at all satisfied = 1 to very satisfied = 7.

## **Socio-economic status**

The household income and parents’ educational attainment were collected form the parental report at children’s age 10 years. We asked annual household income for 11 levels, and then quantified into four categories due to imbalance between levels: less than 2.99 million = 1, 3 to 5.99 million = 2, 6 to 9.99 million = 3, 10 million and over = 4.^1^ Parental educational attainment was obtained using seven levels (junior high school, to high school, to junior college, to vocational school, to 4-year undergraduate school, to 6-year undergraduate, and graduate school), and quantified into four: high school or less =1, 2-year college = 2, 4-year university = 3, graduate university = 4.^1^

# **References**

Achenbach TM. (1991). *Child Behavior Checklist/4-18*. Burlington, VT: University of Vermont.

Ando, S., Nishida, A., Yamasaki, S., Koike, S., Morimoto, Y., Hoshino, A., … Kasai, K. (2019). Cohort Profile: The Tokyo Teen Cohort study (TTC). *International Journal of Epidemiology*, *48*(5), 1414-1414g. https://doi.org/10.1093/ije/dyz033

Angold, A., Costello, E. J., Messer, S. C., Pickles, A. (1995). Development of a short questionnaire for use in epidemiological studies of depression in children and adolescents. *International Journal of Methods in Psychiatric Research*, *5*(4), 237–249.

Costello A, Edelbrock C, Kalas R, Kessler M, K. S. (1982). *NIMH diagnostic interview schedule for children: child version*. Rockville, MD: National Institute of Mental Health.

Goldberg, D. P., & Hillier, V. F. (1979). A scaled version of the General Health Questionnaire. *Psychological Medicine*, *9*(1), 139–145. https://doi.org/10.1017/S0033291700021644

Kelleher, I., Harley, M., Murtagh, A., & Cannon, M. (2011). Are screening instruments valid for psychotic-like experiences? A validation study of screening questions for psychotic-like experiences using in-depth clinical interview. *Schizophrenia Bulletin*, *37*(2), 362–369. https://doi.org/10.1093/schbul/sbp057

Kessler, R. C., Andrews, G., Colpe, L. J., Hiripi, E., Mroczek, D. K., Normand, S. L. T., … Zaslavsky, A. M. (2002). Short screening scales to monitor population prevalences and trends in non-specific psychological distress. *Psychological Medicine*, *32*(6), 959–976. https://doi.org/10.1017/S0033291702006074

Matsuishi, T., Nagano, M., Araki, Y., Tanaka, Y., Iwasaki, M., Yamashita, Y., … Kakuma, T. (2008). Scale properties of the Japanese version of the Strengths and Difficulties Questionnaire (SDQ): a study of infant and school children in community samples. *Brain & Development*, *30*(6), 410–415. https://doi.org/10.1016/j.braindev.2007.12.003

Okada, N., Ando, S., Sanada, M., Hirata-Mogi, S., Iijima, Y., Sugiyama, H., … Kasai, K. (2019). Population-neuroscience study of the Tokyo TEEN Cohort (pn-TTC): Cohort longitudinal study to explore the neurobiological substrates of adolescent psychological and behavioral development. *Psychiatry and Clinical Neurosciences*, *73*(5), 231–242. https://doi.org/10.1111/pcn.12814

Solberg, M. E., & Olweus, D. (2003). Prevalence estimation of school bullying with the Olweus Bully/Victim Questionnaire. *Aggressive Behavior*, *29*(3), 239–268. https://doi.org/10.1002/ab.10047

# **Table S1. Descriptive statistics of TTC at age 10 between boys and girls**

|  |  |  | Boys (n=1684) | | Girls (n=1487) | |  |
| --- | --- | --- | --- | --- | --- | --- | --- |
|  |  |  | Number/*Mean* | %/*SD* | Number/*Mean* | %/*SD* | p^a^ |
| Age (Month) | | | *122.16* | *3.37* | *122.02* | *3.22* | 0.23 |
| Psychological symptoms | | |  |  |  |  |  |
|  | CBCL | Internalized score | *53.51* | *8.80* | *53.41* | *8.64* | 0.74 |
|  |  | Externalized score | *51.50* | *8.52* | *51.30* | *8.47* | 0.51 |
|  | SDQ | Emotional symptom | *1.18* | *0.52* | *1.23* | *0.58* | 0.02 |
|  |  | Conduct problems | *1.24* | *0.58* | *1.17* | *0.50* | <0.01 |
|  |  | Hyperactivity/Inattention | *1.30* | *0.66* | *1.14* | *0.47* | <0.01 |
|  |  | Peer relationship | *1.21* | *0.56* | *1.13* | *0.44* | <0.01 |
|  |  | Prosocial behavior | *1.50* | *0.75* | *1.34* | *0.65* | <0.01 |
|  | SMFQ |  | *5.11* | *4.61* | *4.36* | *4.51* | <0.01 |
|  | APSS |  | *0.95* | *0.95* | *0.79* | *0.86* | <0.01 |
| Bully Involvement | | |  |  |  |  |  |
|  | Bullied | Left out | 257 | 15.9% | 235 | 16.2% | 0.86 |
|  |  | Called mean names | 370 | 23.0% | 225 | 15.5% | <0.01 |
|  |  | Hit lightly | 240 | 14.9% | 111 | 7.7% | <0.01 |
|  |  | Hit strongly | 145 | 9.0% | 49 | 3.4% | <0.01 |
|  |  | Things taken | 55 | 3.4% | 21 | 1.5% | <0.01 |
|  |  | Not bullied | 1025 | 63.6% | 1056 | 73.0% | <0.01 |
|  | Bullying | Left out | 102 | 6.5% | 109 | 7.6% | 0.31 |
|  |  | Called mean names | 140 | 9.0% | 44 | 3.1% | <0.01 |
|  |  |  | Boys (n=1684) | | Girls (n=1487) | |  |
|  |  |  | Number/*Mean* | %/*SD* | Number/*Mean* | %/*SD* | p^a^ |
|  | Bullying | Hit lightly | 100 | 6.4% | 33 | 2.3% | <0.01 |
|  |  | Hit strongly | 33 | 2.1% | 9 | 0.6% | <0.01 |
|  |  | Things taken | 7 | 0.4% | 8 | 0.6% | 0.88 |
|  |  | Not bully | 1290 | 82.8% | 1294 | 89.7% | <0.01 |

Abbreviations: CBCL, the Child Behavior Checklist; SDQ, the Strength and Difficulties Questionnaire; SMFQ, the Short Mood and Feeling Questionnaire; APSS, the Adolescent Psychotic-Like Symptom Screener.

^a^ Paired t-test was performed between boys and girls.

# **Table S2. Descriptive statistics of TTC at age 12 between boys and girls**

|  |  |  | Boys (n=1587) | | Girls (n=1418) | |  |
| --- | --- | --- | --- | --- | --- | --- | --- |
|  |  |  | Number/Mean | %/SD | Number/Mean | %/SD | p^a^ |
| Age (Month) | | | *146.03* | *3.6* | *146.03* | *3.73* | 0.99 |
| Psychological symptoms | | |  |  |  |  |  |
|  | CBCL | Internalized score | *52.09* | *8.98* | *52.23* | *8.6* | 0.69 |
|  |  | Externalized score | *49.52* | *8.53* | *49.81* | *7.9* | 0.36 |
|  | SDQ | Emotional symptom | *1.16* | *0.49* | *1.18* | *0.52* | 0.12 |
|  |  | Conduct problems | *1.22* | *0.56* | *1.13* | *0.47* | <0.01 |
|  |  | Hyperactivity/Inattention | *1.23* | *0.59* | *1.1* | *0.41* | <0.01 |
|  |  | Peer relationship | *1.19* | *0.53* | *1.17* | *0.5* | 0.12 |
|  |  | Prosocial behavior | *1.6* | *0.8* | *1.4* | *0.7* | <0.01 |
|  | SMFQ |  | *3.78* | *4.32* | *3.91* | *4.67* | 0.46 |
|  | APSS |  | *0.81* | *0.97* | *0.86* | *1.01* | 0.18 |
| Involvement of bullying | | |  |  |  |  |  |
|  | Bullied | Left out | 82 | 6.3% | 131 | 11.1% | <0.01 |
|  |  | Called mean names | 160 | 12.2% | 100 | 8.5% | <0.01 |
|  |  | Hit lightly | 77 | 5.9% | 53 | 4.5% | 0.14 |
|  |  | Hit strongly | 31 | 2.4% | 11 | 0.9% | <0.01 |
|  |  | Things taken | 23 | 1.8% | 12 | 1.0% | 0.16 |
|  |  | Not bullied | 1060 | 81.1% | 961 | 81.4% | 0.87 |
|  | Bullying | Left out | 47 | 3.6% | 66 | 5.6% | 0.02 |
|  |  | Called mean names | 67 | 5.1% | 31 | 2.6% | <0.01 |
|  |  |  | Boys (n=1587) | | Girls (n=1418) | |  |
|  |  |  | Number/Mean | %/SD | Number/Mean | %/SD | p^a^ |
|  | Bullying | Hit lightly | 43 | 3.3% | 17 | 1.4% | <0.01 |
|  |  | Hit strongly | 7 | 0.5% | 3 | 0.3% | 0.44 |
|  |  | Things taken | 4 | 0.3% | 3 | 0.3% | 1 |
|  |  | Not bully | 1190 | 90.4% | 1082 | 91.5% | 0.34 |

Abbreviations: CBCL, the Child Behavior Checklist; SDQ, the Strength and Difficulties Questionnaire; SMFQ, the Short Mood and Feeling Questionnaire; APSS, the Adolescent Psychotic-Like Symptom Screener.

^a^ Paired t-test was performed between boys and girls.

# **Table S3. Bifactor analysis of psychological symptoms at age 10**

|  |  |  |  | Factor | | | | |
| --- | --- | --- | --- | --- | --- | --- | --- | --- |
| Questionnaire | No. | Item | Mean (S.D.) | General psychopathology | Depressive symptoms | Aggressive behaviors | Psychotic symptoms | Somatic symptoms |
| CBCL | 50 | Too fearful or anxious | 0.15 (0.27) | **0.620** | -0.224 | -0.035 | -0.002 | 0.012 |
|  | 45 | Nervous or tense | 0.11 (0.22) | **0.595** | -0.180 | 0.026 | -0.022 | 0.063 |
|  | 112 | Worries | 0.14 (0.25) | **0.593** | -0.204 | -0.068 | 0.007 | 0.056 |
|  | 75 | Too shy or timid | 0.15 (0.26) | **0.583** | -0.187 | -0.032 | -0.053 | -0.119 |
|  | 71 | Self-conscious or easily embarrassed | 0.20 (0.29) | **0.555** | -0.148 | 0.011 | -0.059 | -0.080 |
| SDQ | 8 | Many worries of often seems worried | 0.10 (0.22) | **0.516** | -0.037 | 0.056 | -0.038 | 0.103 |
| SMFQ | 8 | Hated self | 0.12 (0.25) | **0.339** | **0.654** | -0.035 | -0.014 | 0.005 |
|  | 11 | Unloved | 0.15 (0.28) | **0.306** | **0.620** | -0.011 | -0.006 | 0.008 |
|  | 5 | Felt no good | 0.15 (0.28) | **0.314** | **0.551** | 0.007 | 0.053 | -0.016 |
|  | 9 | Bad person | 0.12 (0.25) | **0.223** | **0.515** | 0.083 | -0.008 | -0.001 |
|  | 13 | Everything wrong | 0.16 (0.27) | **0.292** | **0.503** | -0.007 | -0.015 | -0.001 |
| SDQ | 5 | Often loses temper | 0.18 (0.27) | **0.297** | -0.011 | **0.752** | -0.013 | 0.003 |
| CBCL | 95 | Temper tantrums or hot temper | 0.27 (0.33) | **0.341** | -0.010 | **0.569** | -0.017 | -0.017 |
|  | 3 | Argues a lot | 0.10 (0.23) | **0.235** | 0.040 | **0.534** | 0.047 | 0.010 |
| APSS | 6 | Visual hallucinations | 0.28 (0.32) | **0.138** | -0.020 | 0.019 | **0.698** | 0.024 |
|  | 4 | Auditory hallucinations | 0.22 (0.34) | **0.137** | -0.005 | -0.036 | **0.620** | -0.018 |
|  | 3 | Spying | 0.36 (0.36) | **0.195** | 0.118 | 0.008 | **0.429** | -0.048 |
| CBCL | 56f | Often complains of headaches, stomachaches or sickness | 0.22 (0.34) | **0.183** | 0.007 | -0.047 | -0.014 | **0.615** |
|  |  |  |  | Factor | | | | |
| Questio-nnaire | No. |  | Mean (S.D.) | General psychopathology | Depressive symptoms | Aggressive behaviors | Psychotic symptoms | Somatic symptoms |
| CBCL | 56c | Headaches | 0.15 (0.27) | **0.158** | -0.013 | -0.006 | -0.007 | **0.578** |
| SDQ | 3 | Stomachaches | 0.04 (0.16) | **0.287** | 0.010 | 0.109 | 0.042 | **0.480** |
|  |  |  | SS loading | 2.92 | 1.86 | 1.21 | 1.10 | 0.99 |

Abbreviations: CBCL, the Child Behavior Checklist; SDQ, the Strength and Difficulties Questionnaire; SMFQ, the Short Mood and Feeling Questionnaire; APSS, the Adolescent Psychotic-Like Symptom Screener.

# **Table S4. Bifactor analysis of psychological symptoms at age 12**

|  |  |  |  | Factor | | | | |
| --- | --- | --- | --- | --- | --- | --- | --- | --- |
| Questionnaire | No. | Item | Mean (S.D.) | General psychopathology | Depressive symptoms | Aggressive behaviors | Psychotic symptoms | Somatic symptoms |
| CBCL | 45 | Nervous or tense | 0.11 (0.23) | **0.636** | -0.077 | 0.046 | -0.040 | 0.030 |
|  | 112 | Worries | 0.11 (0.22) | **0.630** | -0.072 | 0.007 | -0.055 | 0.002 |
|  | 75 | Too shy or timid | 0.12 (0.24) | **0.603** | -0.057 | -0.023 | -0.116 | -0.038 |
|  | 71 | Self-conscious or easily embarrassed | 0.17 (0.27) | **0.570** | -0.015 | 0.048 | -0.114 | -0.024 |
| SDQ | 8 | Many worries of often seems worried | 0.11 (0.23) | **0.560** | 0.046 | 0.029 | -0.032 | 0.111 |
| CBCL | 52 | Feels too guilty | 0.05 (0.17) | **0.527** | 0.005 | 0.000 | -0.040 | 0.010 |
| SMFQ | 8 | Hated self | 0.14 (0.28) | **0.240** | **0.734** | -0.028 | -0.003 | 0.006 |
|  | 11 | Unloved | 0.13 (0.27) | **0.227** | **0.711** | 0.021 | 0.006 | -0.008 |
|  | 12 | Never be as good | 0.12 (0.26) | **0.196** | **0.711** | 0.020 | -0.002 | 0.031 |
|  | 13 | Everything wrong | 0.11 (0.24) | **0.228** | **0.641** | -0.016 | 0.000 | -0.023 |
|  | 5 | Felt no good | 0.11 (0.25) | **0.237** | **0.635** | 0.002 | 0.003 | -0.022 |
| CBCL | 95 | Temper tantrums or hot temper | 0.07 (0.19) | **0.269** | -0.010 | **0.712** | 0.003 | -0.010 |
|  | 68 | Screams a lot | 0.05 (0.16) | **0.294** | 0.008 | **0.633** | 0.006 | 0.007 |
|  | 22 | Disobedient at home | 0.23 (0.29) | **0.250** | 0.021 | **0.432** | -0.035 | 0.026 |
| APSS | 4 | Auditory hallucinations | 0.22 (0.33) | **0.167** | -0.014 | 0.006 | **0.775** | -0.021 |
|  | 6 | Visual hallucinations | 0.17 (0.33) | **0.172** | -0.012 | -0.011 | **0.667** | 0.023 |
|  | 3 | Spying | 0.14 (0.29) | **0.113** | 0.116 | 0.000 | **0.463** | 0.031 |
| CBCL | 56b | Headaches | 0.06 (0.18) | **0.257** | 0.012 | 0.006 | -0.010 | **0.624** |

|  |  |  |  | Factor | | | | |
| --- | --- | --- | --- | --- | --- | --- | --- | --- |
| Questionnaire | No. | Item | Mean (S.D.) | General psychopathology | Depressive symptoms | Aggressive behaviors | Psychotic symptoms | Somatic symptoms |
| SDQ | 3 | Often complains of headaches, stomachaches or sickness | 0.15 (0.28) | **0.289** | -0.030 | 0.011 | 0.022 | **0.522** |
| CBCL | 56c | Nausea, feels sick | 0.02 (0.11) | **0.252** | 0.005 | -0.036 | -0.004 | **0.437** |
|  |  |  | SS loading | 2.84 | 2.41 | 1.10 | 1.31 | 0.87 |

Abbreviations: CBCL, the Child Behavior Checklist; SDQ, the Strength and Difficulties Questionnaire; SMFQ, the Short Mood and Feeling Questionnaire; APSS, the Adolescent Psychotic-Like Symptom Screener.

# **Table S5. Factor analysis of environmental factors at age 10**

|  |  | Factor | | | | | |
| --- | --- | --- | --- | --- | --- | --- | --- |
| Item | Mean (S.D.) | Parental depressive symptom | Bullied | Life  satisfaction | Bullying | Warm parenting style | Socio-economic status |
| K6 No.4 (depressed) | 0.60 (0.78) | **0.822** | 0.073 | -0.130 | 0.068 | -0.127 | -0.063 |
| K6 No.2 (hopeless) | 0.27 (0.62) | **0.730** | 0.082 | -0.117 | 0.052 | -0.104 | -0.079 |
| K6 No.5 (everything an effort) | 0.66 (0.78) | **0.683** | 0.073 | -0.116 | 0.062 | -0.118 | -0.023 |
| K6 No.6 (worthless) | 0.38 (0.73) | **0.674** | 0.079 | -0.107 | 0.023 | -0.153 | -0.051 |
| K6 No.3 (restless) | 0.40 (0.66) | **0.650** | 0.065 | -0.074 | 0.062 | -0.084 | -0.059 |
| K6 No.1 (nervous) | 0.63 (0.85) | **0.637** | 0.063 | -0.076 | 0.039 | -0.072 | -0.092 |
| Called mean names | 0.19 (0.40) | 0.080 | **0.725** | -0.214 | 0.370 | -0.020 | -0.011 |
| Left out | 0.16 (0.37) | 0.072 | **0.608** | -0.199 | 0.310 | -0.039 | -0.018 |
| Bullied (school) | 1.50 (1.08) | 0.068 | **0.584** | -0.214 | 0.438 | -0.047 | -0.085 |
| Weakly hit | 0.11 (0.32) | 0.034 | **0.457** | -0.183 | 0.248 | -0.047 | -0.017 |
| Self | 5.43 (1.56) | -0.089 | -0.218 | **0.802** | -0.127 | 0.183 | 0.021 |
| Appearance | 4.89 (1.52) | -0.097 | -0.206 | **0.761** | -0.131 | 0.153 | 0.069 |
| Study | 5.28 (1.45) | -0.093 | -0.197 | **0.556** | -0.160 | 0.076 | 0.069 |
| Friends | 6.17 (1.21) | -0.097 | -0.284 | **0.492** | -0.122 | 0.076 | 0.061 |
| Bullying (school) | 1.14 (0.55) | 0.035 | 0.352 | -0.118 | **0.778** | -0.035 | -0.029 |
| Leave out | 0.07 (0.26) | 0.039 | 0.361 | -0.137 | **0.508** | -0.061 | 0.015 |
| Call mean names | 0.06 (0.24) | 0.059 | 0.416 | -0.120 | **0.488** | -0.043 | -0.043 |
| Bullying (outside school) | 1.05 (0.31) | 0.036 | 0.231 | -0.105 | **0.453** | -0.021 | -0.072 |
| Tell love | 3.00 (0.93) | -0.054 | -0.014 | 0.107 | -0.013 | **0.825** | 0.094 |

|  |  | Factor | | | | | |
| --- | --- | --- | --- | --- | --- | --- | --- |
| Item | Mean (S.D.) | Parental depressive symptom | Bullied | Life  satisfaction | Bullying | Warm parenting style | Socio-economic status |
| Praise | 3.04 (0.79) | -0.099 | -0.085 | 0.126 | -0.044 | **0.760** | 0.111 |
| Talk | 3.00 (0.80) | -0.099 | -0.025 | 0.090 | -0.046 | **0.340** | -0.004 |
| Father education | 2.63 (0.92) | -0.040 | -0.046 | 0.074 | -0.065 | 0.011 | **0.732** |
| Income | 2.96 (0.86) | -0.081 | -0.032 | 0.070 | -0.034 | 0.121 | **0.555** |
| Mother education | 2.26 (0.77) | -0.043 | -0.013 | 0.021 | -0.025 | 0.025 | **0.545** |
|  | SS loadings | 2.96 | 1.78 | 1.54 | 1.40 | 1.32 | 1.16 |

Abbreviations: K6, the Kessler Psychological Distress Scale.

# **Table S6. Factor analysis of environmental factors at age 12**

|  |  | Factor | | | | |
| --- | --- | --- | --- | --- | --- | --- |
| Item | Mean (S.D.) | Parental depressive symptom | Life  satisfaction | Bullied | Bullying | Warm parenting style |
| GHQ No.25 (make away with yourself) | 1.29 (0.62) | **0.890** | -0.082 | 0.069 | 0.016 | -0.128 |
| GHQ No.27 (dead and away from it all) | 1.15 (0.47) | **0.868** | -0.109 | 0.079 | 0.013 | -0.112 |
| GHQ No.24 (life not worth living) | 1.29 (0.56) | **0.832** | -0.105 | 0.057 | 0.028 | -0.156 |
| GHQ No.28 (idea of taking your life) | 1.10 (0.35) | **0.748** | -0.053 | 0.075 | -0.021 | -0.092 |
| GHQ No.26 (nerves too bad) | 1.20 (0.51) | **0.730** | -0.070 | 0.099 | 0.046 | -0.114 |
| GHQ No.22 (thinking of yourself as worthless) | 1.37 (0.63) | **0.728** | -0.098 | 0.053 | 0.026 | -0.131 |
| Self | 4.97 (1.60) | -0.093 | **0.866** | -0.206 | -0.094 | 0.155 |
| Appearance | 4.53 (1.50) | -0.099 | **0.811** | -0.189 | -0.105 | 0.152 |
| Study | 5.18 (1.54) | -0.036 | **0.444** | -0.095 | -0.080 | 0.091 |
| Bullied | 1.20 (0.72) | 0.069 | -0.124 | **0.726** | 0.355 | -0.004 |
| Called mean names | 0.10 (0.30) | 0.019 | -0.135 | **0.638** | 0.277 | -0.010 |
| Left out | 0.08 (0.28) | 0.040 | -0.137 | **0.540** | 0.229 | -0.012 |
| Bullied (parent response) | 1.18 (0.62) | 0.080 | -0.124 | **0.460** | 0.089 | 0.000 |
| Bullying | 1.08 (0.42) | 0.051 | -0.115 | 0.252 | **0.881** | -0.067 |
| Leave out | 0.05 (0.21) | 0.010 | -0.072 | 0.191 | **0.515** | 0.013 |
| Call mean names | 0.04 (0.19) | -0.005 | -0.062 | 0.232 | **0.433** | -0.002 |
| Praise | 2.87 (0.78) | -0.064 | 0.141 | -0.006 | -0.003 | **0.820** |
| Tell love | 2.78 (0.96) | -0.032 | 0.111 | 0.005 | 0.000 | **0.747** |
| Relation | 3.35 (0.66) | -0.140 | 0.098 | -0.009 | -0.024 | **0.356** |

|  |  | Factor | | | | |
| --- | --- | --- | --- | --- | --- | --- |
|  |  | Parental depressive symptom | Life  satisfaction | Bullied | Bullying | Warm parenting style |
|  | SS loadings | 3.88 | 1.60 | 1.42 | 1.38 | 1.29 |

Abbreviations: GHQ-28, the 28-item version of the General Health Questionnaire.

# **Figure S1. Correlation matrix between psychological symptom scores at ages 10 and 12.**


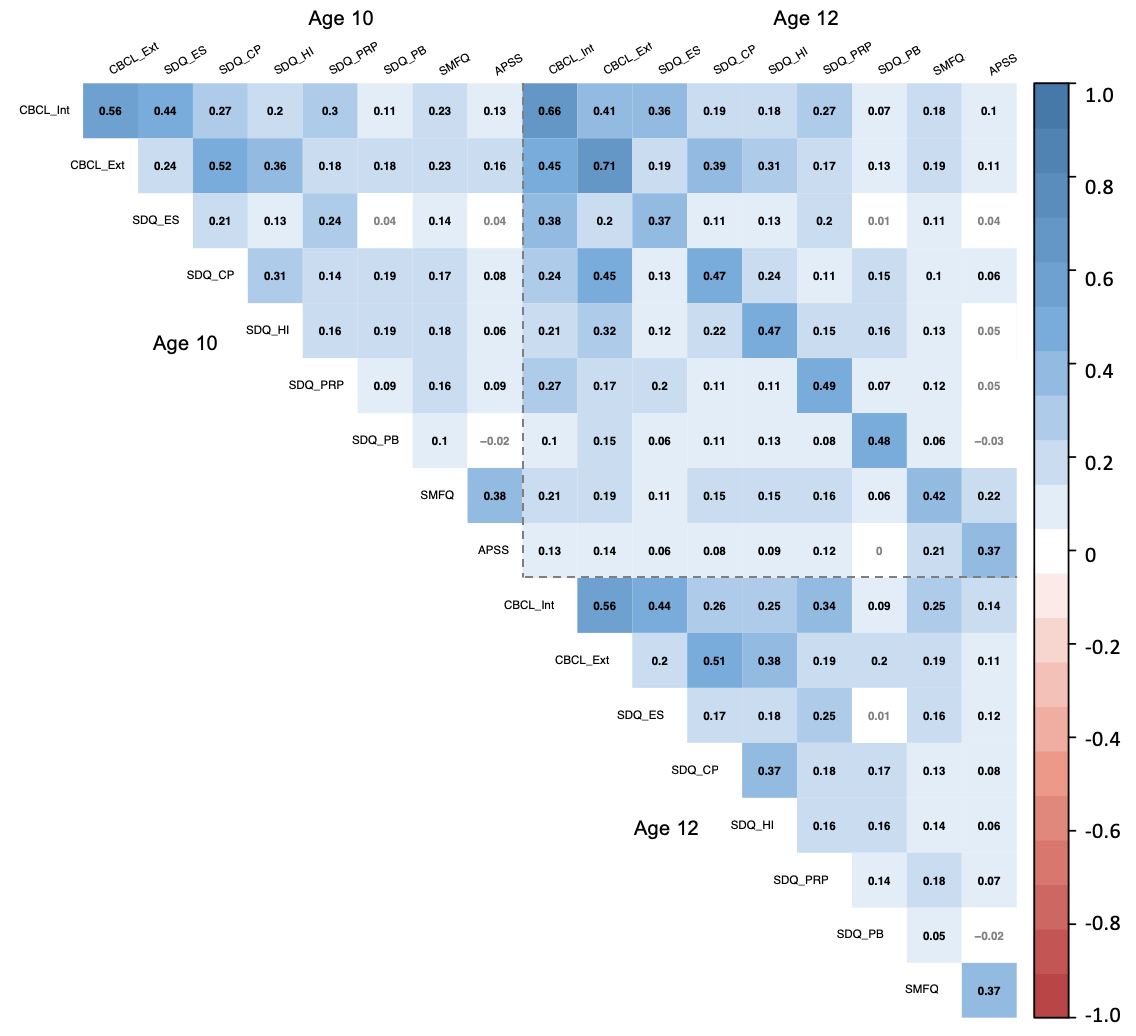


Non-significant correlation scores were shown in grey color. Dot lines indicates the borderlines between symptom scores at ages 10 and 12.

Abbreviations: CBCL, the Child Behavior Checklist; Int, Internalized Score; Ext, Externalized Score; SDQ, the Strength and Difficulties Questionnaire; ES, Emotional Symptoms; CP, Conduct Problems; HI, Hyperactivity-Inattention; PRP, Peer Relationship; PB, Prosocial Behaviors; SMFQ, the Short Mood and Feeling Questionnaire; APSS, the Adolescent Psychotic-Like Symptom Screener.

# **Figure S2. Scree plot of psychological symptoms at ages 10 and 12.**


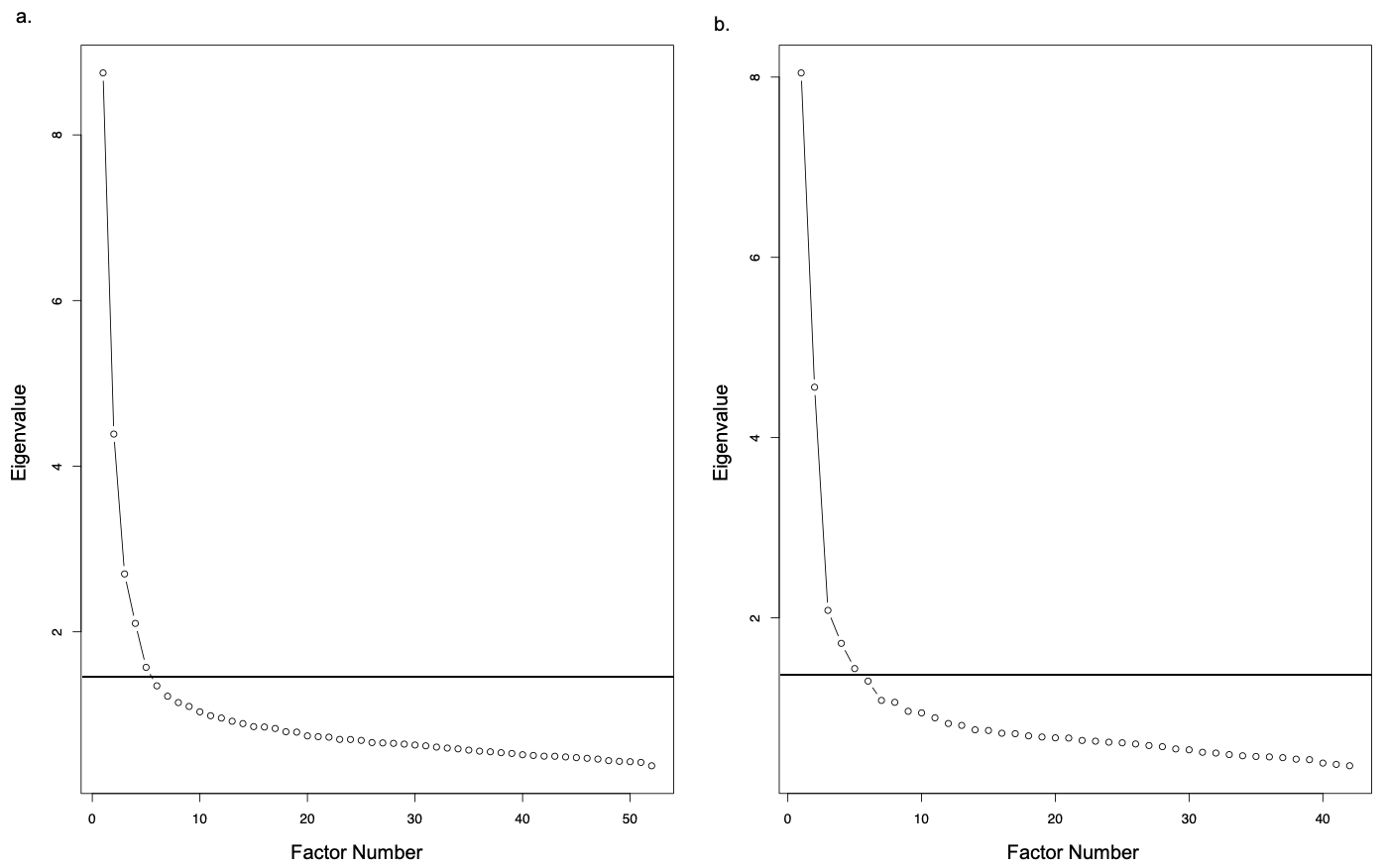
We set eigenvalue to the same number between ages 10 (A) and 12 (B). The black lines indicate the cut point.

**B**

**A**

# **Figure S3. Scree plot of environmental factors at ages 10 and 12.**


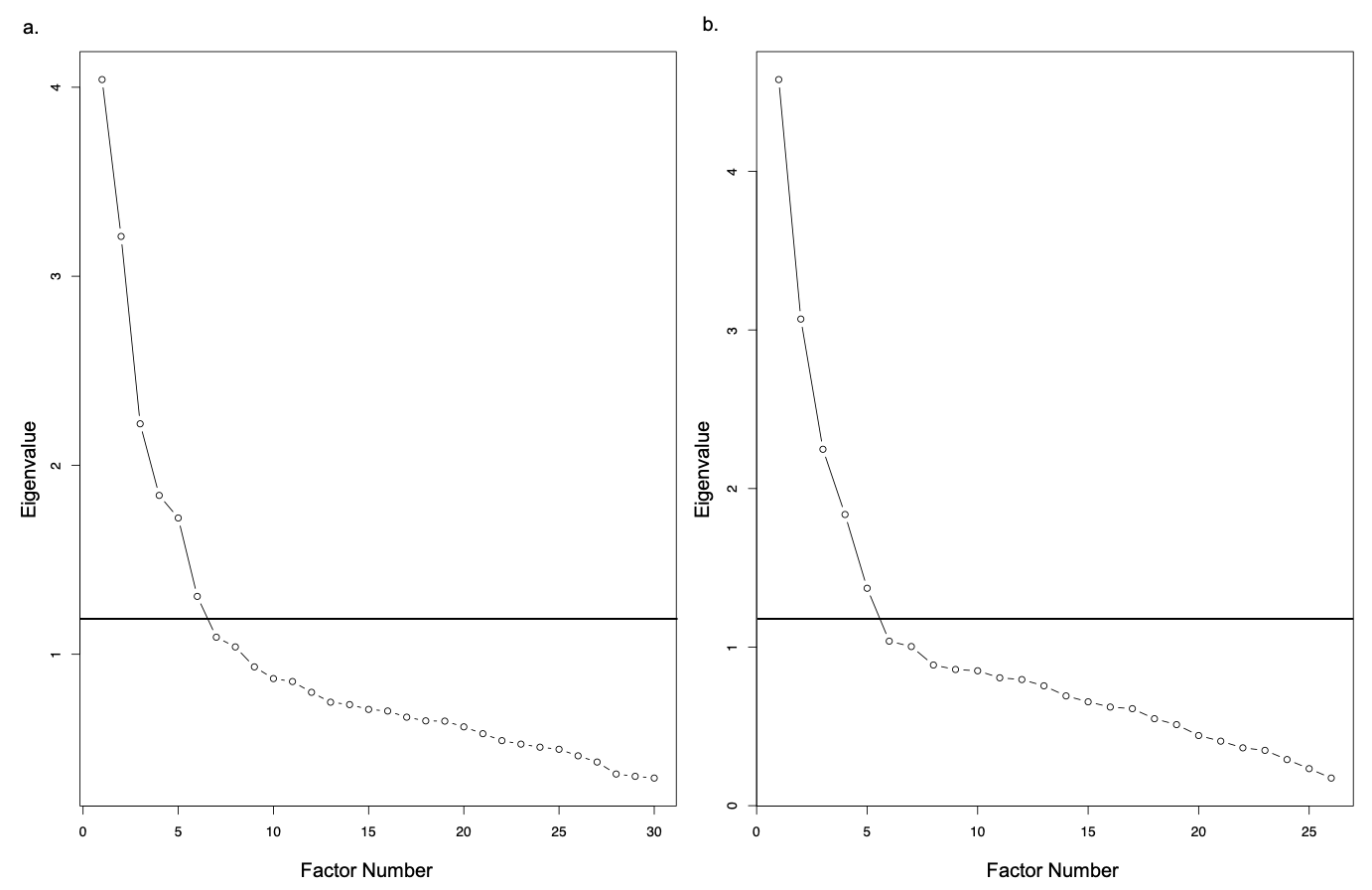
We set eigenvalue to the same number between ages 10 (A) and 12 (B). The black lines indicate the cut point.

**B**

**A**
